# Two Siblings Homozygous for F508del-CFTR Have Varied Disease Phenotypes and Protein Biomarkers

**DOI:** 10.3390/ijms22052631

**Published:** 2021-03-05

**Authors:** Zhihong Zhang, Jin Wang, Yanhui H. Zhang, Tonia E. Gardner, Elizabeth A. Fitzpatrick, Weiqiang Zhang

**Affiliations:** 1 Department of Pediatrics, College of Medicine, University of Tennessee Health Science Center, Memphis, TN 38163, USA; zhangzhihope@126.com; 2 Department of Microbiology, Immunology, and Biochemistry, College of Medicine, University of Tennessee Health Science Center, Memphis, TN 38163, USA; jwang141@uthsc.edu; 3 Department of Bioscience Research, College of Dentistry, University of Tennessee Health Science Center, Memphis, TN 38163, USA; yzhang36@uthsc.edu; 4 University of Tennessee Cystic Fibrosis Care and Research Center at Le Bonheur Children’s Hospital-Methodist University Hospital, Memphis, TN 38103, USA; 5 Departments of Physiology, College of Medicine, University of Tennessee Health Science Center, Memphis, TN 38163, USA; 6 Children’s Foundation Research Institute, Le Bonheur Children’s Hospital, Memphis, TN 38103, USA

**Keywords:** cystic fibrosis (CF), CFTR, F508del-CFTR, disease phenotypes, infection, inflammation, biomarkers

## Abstract

Two siblings with CF are homozygous for F508del (referred to as Subject A and Subject B). Despite having the same CFTR genotype and similar environment, these two subjects exhibited different disease phenotypes. We analyzed their medical records and CF Foundation Registry data and measured inflammatory protein mediators in their sputum samples. Then, we examined the longitudinal relationships between inflammatory markers and disease severity for each subject and compared between them. Subject A presented a more severe disease than Subject B. During the study period, Subject A had two pulmonary exacerbations (PEs) whereas Subject B had one mild PE. The forced expiratory volume in 1 s (FEV_1_, % predicted) values for Subject A were between 34–45% whereas for Subject B varied between 48–90%. Inflammatory protein mediators associated with neutrophils, Th1, Th2, and Th17 responses were elevated in sputum of Subject A compared with Subject B, and also in samples collected prior to and during PEs for both subjects. Neutrophilic elastase (NE) seemed to be the most informative biomarkers. The infectious burden between these two subjects was different.

## 1. Introduction

Cystic fibrosis (CF) is a life-shortening autosomal recessive disease caused by the absence or dysfunction of the CF transmembrane conductance regulator (CFTR) channel activity, resulting from mutations in the *CFTR* gene [1,2]. There are more than 70,000 patients with CF worldwide [3]. The incidence of CF and the frequency of specific mutations vary among ethnic populations [4,5]. Clinically, CF can affect multiple organs, including the upper airway, lungs, pancreas, sweat glands, gastrointestinal (GI) tract, liver, and vas deferens [6]. Chronic lung disease causes most of the CF-associated morbidity and mortality. CF lung disease starts early and silently in infancy and progresses rapidly during childhood. CF lung disease is characterized by mucus obstruction, chronic bacterial infection, and excessive inflammation. These symptoms cause bronchiectasis and progressive lung function decline [7]. The median life expectancy for patients with CF has improved to more than 40 years, attributable to the early diagnosis, care provided by specialized centers, and development of novel therapies [8]. 

Currently, 2103 mutations have been identified in the *CFTR* gene [9]. Based on defect(s) nature, mutations can be grouped into six categories [10,11]. The classification of CFTR mutations helps define dysfunction-specific strategies to restore CFTR channel function. F508del (cDNA Name: c.1521_1523delCTT; Protein name: p.Phe508del) is the most prevalent CFTR mutation, with approximately 80–85% of CF patients carrying it on at least one allele worldwide. It should be noted that, in addition to genotype, other genetic and environmental factors strongly influence the severity of CF disease, which include modifier genes and genomic regions (e.g., SLC9A3, MUC4/MUC20) [12], socioeconomic status [13], and secondhand smoke exposure [14]. Chronic microbial colonization and infections are also major factors affecting the disease outcomes. Bacteria are considered classic CF pathogens. *Pseudomonas aeruginosa,* methicillin-resistant *Staphylococcus aureus* (MRSA), and *Burkholderia cepacia* complex species have significant impacts on lung function and mortality rates in CF patients. Viral infections (e.g., respiratory syncytial virus) and fungal infections (e.g., *Aspergillus* species) are increasingly recognized as important components of the CF airway infections and contribute to CF disease progression [15]. 

Biomarkers in CF can be categorized into two groups (i) biomarkers of CFTR function, including sweat chloride levels, nasal potential difference, intestinal current measurements, and (ii) biomarkers of disease outcomes, such as biomarkers in blood, biomarkers of inflammation, and biomarkers of infection. Biomarkers in CF are usually measured in broncho-alveolar lavage (BAL) fluid, sputum, exhaled breath, and blood samples [16,17]. Effective CF biomarkers can aid in the disease diagnosis, monitoring, and treatment.

In this study, we show the clinical case of two siblings homozygous for F508del. Despite having the same CFTR genotype and similar environmental exposure, the two siblings exhibited different disease phenotypes. To better understand these disease variations and to potentially identify their clinically relevant biomarkers, we tested a panel of inflammatory protein mediators in their sputum samples and explored the possible correlations between the levels of these mediators and disease severity for each subject. We also compared these parameters between two siblings. 

## 2. Results

### 2.1. Clinical Description

The study subjects are females and 364 days apart in age. They live in the same home environment. The sweat chloride level of Subject A at the age of 5 months and 16 days was 115 mmol/L (right arm). The sweat chloride levels for Subject B, the younger sibling, at the age of 12 days were 102.1 mmol/L (right arm) and 99.3 mmol/L (left arm). Genotyping results showed that they were both homozygous for F508del. 

Before this study, Subject A has had an average of 6–8 hospitalizations per year for pulmonary exacerbations (PEs) or other CF complications since she was diagnosed with CF. She has consistently grown MRSA in cultures for over ten years. Subject B had her first hospitalization at age 6 years. She then had 1–2 hospitalizations for PEs and other CF complications until age 11. Since age 11 years, she has had an average of 5–7 hospitalizations per year. She has intermittently grown MRSA in cultures for over ten years prior to the study. 

This study began collecting samples when Subjects A and B were 15 years of age. During the study period, Subject A was prescribed the standard of care therapies for CF including albuterol twice daily, 7% hypertonic saline twice daily, dornase alpha daily, azithromycin three times weekly. She was instructed to complete airway clearance with high frequency chest wall oscillation twice daily when well and increase to four times daily when sick. She was also taking Orkambi^®^ (Lumacaftor/Ivacaftor) the only commercially available CFTR modifiers for her age and genotype at that time. Her adherence to therapies over time was estimated to be moderate. Subject B also received the standard of care therapies for CF, including albuterol twice daily, 7% hypertonic saline twice daily, dornase alpha daily, azithromycin three times weekly and cycled inhaled antibiotics (targeting *Pseudomonas aeruginosa*). She was instructed to complete airway clearance with high frequency chest wall oscillation twice daily when well and increase to four times daily when sick. Her adherence to therapies over time was estimated to be moderate. Subject B was not taking CFTR modulators at the time of this study due to drug interactions with concomitant non-CF medications she was prescribed. 

In this study, we investigated the disease features of these two siblings during clinic visits, with an emphasis on lung disease presentations. We also collected sputum samples during these visits to test a panel of inflammatory protein mediators. For Subject A, these visits started in November 2015 and ended in June 2017 and contained seven visits (referred to as Visit 1–7 in this paper). For Subject B, these visits started in August 2016 and ended in June 2017 and contained five visits (referred to as Visit 1–5 in this paper). The detailed information of these visits is provided in the Materials and Methods section. 

Subject A generally had more severe lung disease manifestation than Subject B. Subject A had two PEs, which occurred 6 days after Visit 1, and five days after Visit 3. In contrast, Subject B had only one mild exacerbation at Visit 1. The forced expiratory volume in 1 s (FEV_1_, % predicted) values for Subject A were between 34–45%. For Subject B, FEV_1_ (% predicted) varied from 48–90%, with the most recent sample at 90% (Table 1). Subject A had bilateral inspiratory crackles on exams. Subject B’s lungs were clear on exams except that crackles were heard at the right base at Visit 4. Despite living in the same home, distinct microbes were isolated from the sputum of each sibling. Subject A routinely tested positive for MRSA and *Aspergillus fumigatus* and occasionally for *Stenotrophomonas maltophilia*. Sputum from Subject B exhibited more variations in the microbes isolated that included *Pseudomonas aeruginosa*, MRSA, *stenotrophomonas*, *Candida albicans*, *Escherichia coli* and *Klebsiella pneumoniae* (Table 2 and Table 3). Other major symptoms of these siblings are summarized in Table 2 and Table 3. 

### 2.2. Levels of Neutrophil-Associated Markers in Sputum Samples

**Figure 1:**
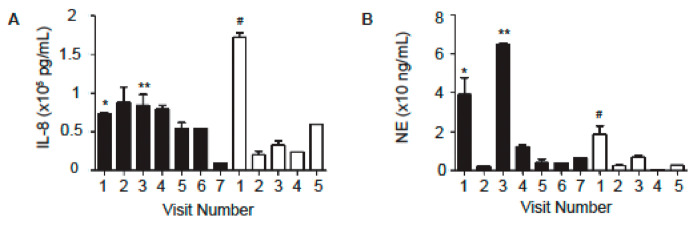
IL-8 and NE levels in the sputum samples from Subject A (black bars) and Subject B. (**A**) IL-8 levels. (**B**) NE levels. The samples were measured using ELISA kits and the data are presented as Mean ± standard error of mean (SEM). *n* = 4–6. * admitted into the emergency room (ER) 6 days later for pulmonary exacerbation (PE); ** admitted into ER 5 days later for PE; ^#^ admitted into ER for PE on this visit.

Because inflammation in CF lungs is dominated by the infiltration of neutrophils and the release of oxidants and proteases [18,19], we first measured interleukin 8 (IL-8) and neutrophilic elastase (NE) levels in these samples using enzyme-linked immunosorbent assay (ELISA). IL-8 is a proinflammatory and chemoattractant cytokine, and has been shown to play a key role in the recruitment of neutrophils and other immune cells during inflammation [19]. Subject A generally had higher IL-8 levels in sputum samples than Subject B (Figure 1A, Table 4). Also, the NE levels of Subject A were generally higher than those of Subject B (Figure 1B, Table 4). However, these differences did not reach the level of statistical significance (*p* = 0.97 for IL-8; *p* = 0.22 for NE). During PE period of Subject B and preceding the PEs of Subject A, the IL-8 and NE levels increased dramatically (Figure 1, Table 4). The NE levels marginally correlated with FEV_1_ (% predicted) for Subject A (*p* = 0.08 (Pearson’s product-moment correlation and Spearman’s rank correlation)), but not for Subject B. There was no correlation between the IL-8 level and FEV_1_ (% predicted) for either subject. 

**Figure 2:**
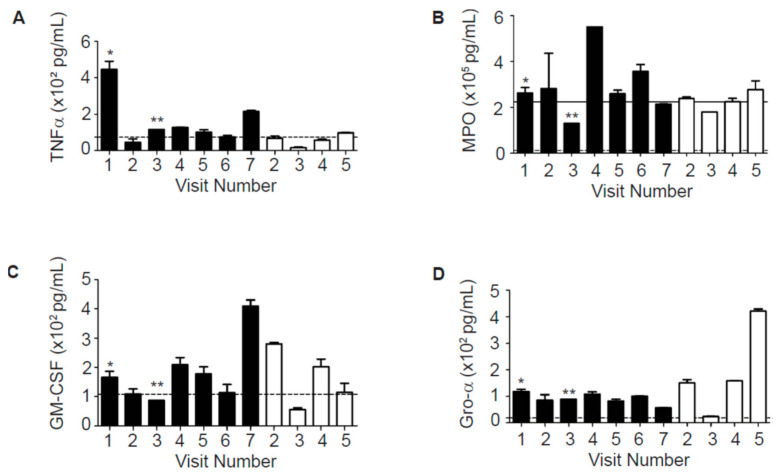
The levels of neutrophil-associated markers, (**A**) TNFα, (**B**) MPO, (**C**) GM-CSF, and (**D**) Gro-α in the sputum samples from Subject A (black bars) and Subject B. Cytokines were measured in sputum samples in duplicate using a bead-based multiplex assay. The dotted lines indicate the lower limit of detection for each specific cytokine; the solid line indicates the upper limit of detection for each specific cytokine. * admitted into the emergency room (ER) 6 days later for pulmonary exacerbation (PE); ** admitted into ER 5 days later for PE.

Based on IL-8 and NE data, which seemed to show higher concentrations of these cytokines in the sputum of Subject A who exhibited a more severe lung disease phenotype, we next measured a panel of other inflammatory mediators in these samples using a multiplex bead-based assay. We found that the neutrophil myeloperoxidase (MPO) and Tumor necrosis factor alpha (TNFα) were generally increased in Subject A in comparison to Subject B (Figure 2A,B). TNFα peaked preceding the 1st PE of Subject A (sample from Visit 1) (Figure 2A). Granulocyte-macrophage colony-stimulating factor (GM-CSF) was elevated in samples from both patients (Figure 2C). Interestingly, Subject B had increased levels of the neutrophil chemokine growth related α protein (Gro-α) compared to Subject A (Figure 2D). 

### 2.3. Levels of Other Inflammatory Mediators in Sputum Samples

**Figure 3:**
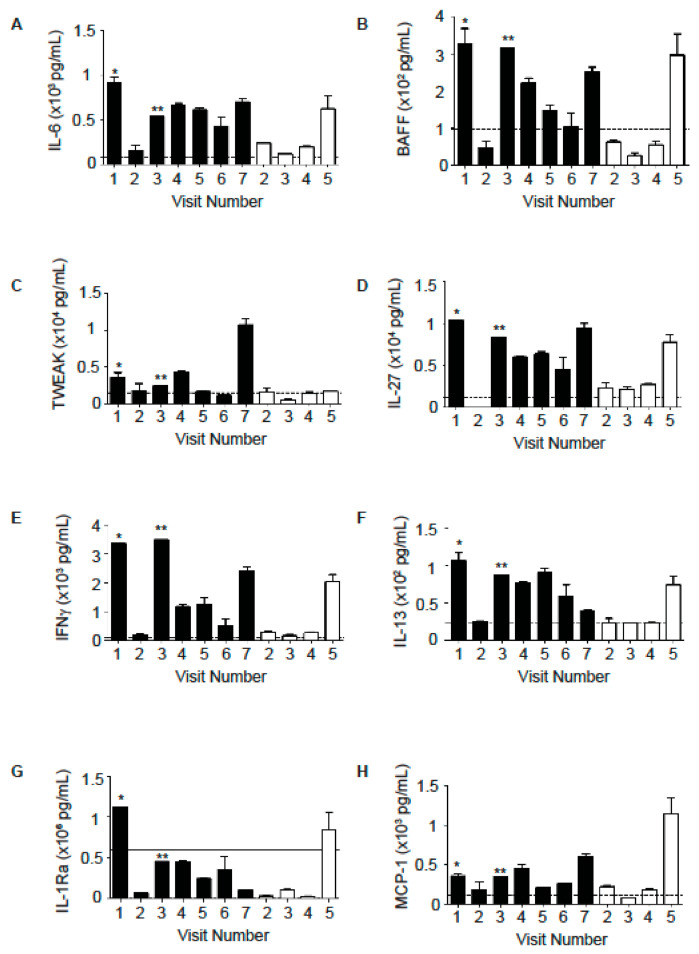
Inflammatory mediators in sputum samples from Subject A (black bars) were increased in comparison to Subject B. (**A**) IL-6 levels. (**B**) BAFF levels. (**C**) TWEAK levels. (**D**) IL-27 levels. (**E**) IFNγ levels. (**F**) IL-13 levels. (**G**) IL-1Ra levels. (**H**) MCP-1 levels. Cytokines were measured in sputum samples in duplicate using a bead-based multiplex assay. The dotted lines indicate the lower limit of detection for each specific cytokine; the solid line indicates the upper limit of detection for each specific cytokine. * admitted into the emergency room (ER) 6 days later for pulmonary exacerbation (PE); ** admitted into ER 5 days later for PE.

**Figure 4:**
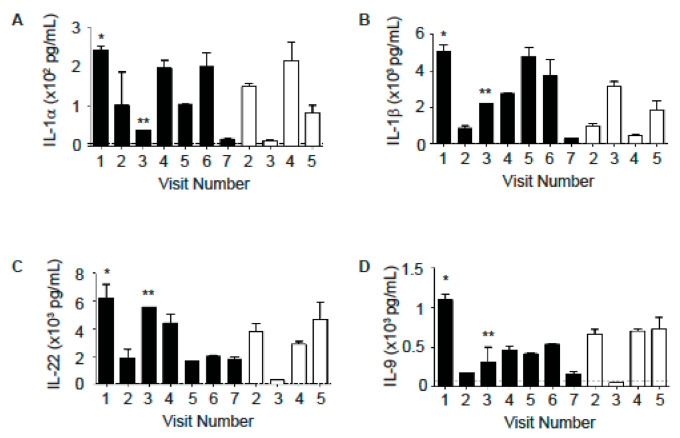
Cytokines that were elevated in both subjects. Subject A (black bars). (**A**) IL-1α levels. (**B**) IL-1β levels. (**C**) IL-22 levels. (**D**) IL-9 levels. Cytokines were measured in sputum samples in duplicate using a bead-based multiplex assay. The dotted lines indicate the lower limit of detection for each specific cytokine. * admitted into the emergency room (ER) 6 days later for pulmonary exacerbation (PE); ** admitted into ER 5 days later for PE.

In addition to neutrophil-associated markers, CF patients also exhibit elevated levels of other pro-inflammatory cytokines. We found that several cytokines including IL-6, B-cell activating factor (BAFF), serum TNF-related weak inducer of apoptosis (TWEAK), IL-27, interferon gamma (IFNγ), IL-13, monocyte chemoattractant protein-1(MCP-1), and IL-1 receptor antagonist (IL-1Ra) were increased in the sputum of Subject A in comparison to Subject B (Figure 3A–H). Only during a GI illness did Subject B exhibit an increase in these cytokines (sample from Visit 5, Figure 3A–H). There were other cytokines, including IL-22, IL-9, IL-1α, and IL-1β, that were elevated in sputum from both patients, although their levels fluctuated between samples collected at different time points (Figure 4A–D). 

## 3. Discussion

In CF clinical trials, three types of outcome measures are used: clinical end-points, surrogate end-points, and biomarkers [16]. The major clinical end-points include survival, the frequency of respiratory exacerbation, and quality of life. FEV_1_ is the most commonly used surrogate end-point. The commonly used biomarkers include inflammatory markers, sputum bacterial density, and mucocilliary clearance [16]. Some of these outcome measures are also used in CF diagnosis and management. Because early detection and intervention are key to improving long-term outcomes in CF, there is an urgent need to identify accurate, non-invasive, and clinically relevant biomarkers to aid in the diagnosis, monitoring of disease progression, treatment decisions, and measures of outcome in clinical trials [17,20].

Sputum IL-8 and NE levels have been found to be negatively associated with lung function in CF and correlate with disease severity [21,22]. In our study, Subject A generally had more severe lung disease manifestation than Subject B (Table 1, Table 2 and Table 3). The average NE and IL-8 levels in sputum samples from Subject A were higher than those from Subject B, with a 2.1-fold increase of NE level for subject A (Table 4).

In a prospective, longitudinal cohort study in children with CF, Sagel and colleagues examined the relationships between sputum biomarkers and lung function. They found that changes in sputum biomarkers of inflammation and proteolysis were related to changes in lung function. The declines in FEV_1_ (% predicted) were found to significantly associate with increases in neutrophil counts, NE, and IL-1β, and marginally associate with decreases in NE antiprotease complexes and secretory leukoprotease inhibitor and with elevation in IL-8. Sputum NE was found to be the most informative biomarker to monitor disease activity [22]. In our study, NE showed a tendency to associate with disease severity in both subjects. For Subject B, NE level peaked during exacerbation (sample from Visit 1, Figure 1B, Table 4). NE levels also increased nine-fold for Subject A preceding two exacerbations (Samples from Visits 1 and 3, Figure 1B, Table 4). We attempted the correlation study and found that NE levels marginally correlated with FEV_1_ (% predicted) for Patent A [*p* = 0.08 (Pearson’s product-moment correlation and Spearman’s rank correlation)], but not for Subject B. Correlation was not found between the IL-8 levels and FEV_1_% for Patent A or for Subject B. The lack of statistical significance in some of the analyses could be due to the small sample size.

Sputum MPO levels from CF subjects were found inversely correlated with FEV_1_% predicted [23]. In our study, MPO and TNFα were generally increased in Subject A in comparison to Subject B (Figure 2A,B).

We also tested the samples for a panel of cytokines associated with Th1, Th2 or Th17 responses. Subject A showed increases in cytokines associated with a Th17 response (IL-6, BAFF and TWEAK), cytokines associated with a Th1 response (IFNγ, IL-27, BAFF) and a Th2 response (IL-13) in comparison to Subject B (Figure 3). BAFF is a cytokine that is critical for B cell survival [24,25,26] and has more recently been shown to promote both Th1 and Th17 responses [27,28] and contribute to lung pathology in a murine chronic obstructive pulmonary disease model [29]. Interestingly, Subject A also had higher levels of the anti-inflammatory protein IL-1Ra in comparison to Subject B. Several cytokines (IL-22, IL-9, IL-1α, and IL-1β) were elevated in sputum from both patients (Figure 4). The overall increase in cytokine levels in Subject A suggests the activation of multiple arms of the immune system in this subject, which may contribute to lung pathology and decreased FEV_1_. This activation may be due to the numbers and types of pathogens that Subject A was exposed to. These patients were siblings who lived in the same household. However, they did not share the same infectious burden. For example, *Aspergillus fumigatus* was frequently isolated from Subject A but not from Subject B. *Pseudomonas aeruginosa* and *Candida albicans* were seen in samples from Subject B but not from Subject A (Table 2 and Table 3). Subject A has consistently grown MRSA in cultures for over ten years. Subject B exhibited a greater diversity of microbes including *Pseudomonas aeruginosa*, MRSA, *Stenotrophomonas maltophilia*, *Candida albicans*, *Escherichia coli*, and *Klebsiella pneumoniae* (Table 2 and Table 3). Additionally, the increased severity of Subject A’s lung disease may result in higher levels of damage-associated molecular patterns (DAMP) that can also contribute to and perpetuate the inflammatory cytokine production.

PEs are known to accelerate the decline of lung function in CF patients. Sputum club cell secretory protein has been shown to negatively associate with CF PE and sputum neutrophil elastase level [30]. Solomon and colleagues found that CF patients with acute PEs had elevated IFNγ-induced protein 10 kDa (IP-10) in nasal lavage fluid, which decreased significantly following antimicrobial therapy. They also found in CF BAL fluid IP-10 was elevated [31]. We did not measure IP-10, however IFNγ, which induces IP-10, was the highest in Subject A preceding the PE (Figure 3E). We found that IL-8 and NE levels increased prior to and during PE for both patients (Figure 1, Table 4).

During our study period, Subject A was taking Orkambi^®^ while Subject B was not on it because of drug-drug interaction issues. Even with Orkambi^®^ in her treatment, Subject A still had the more severe lung disease. The differences in disease severity seen in these subjects could be due to multiple reasons: (i) the microbial infections could play a major role. The infectious burden between these two subjects was different and it is conceivable that an increase in the level and type of microbes in Subject A drove a feed-forward loop of inflammation due to lung damage and DAMP release. Subject A routinely tested positive for MRSA and *Aspergillus fumigatus* whereas Subject B did not. Chronic MRSA infection has been associated with detrimental clinical outcomes in CF patients. Ren and colleagues reported that compared to patients with methicillin sensitive *Staphylococcus aureus* only, patients with MRSA only had significantly lower lung function and increased hospitalization and antibiotic use [32]. Dasenbrook et al. reported that persistent MRSA respiratory infection in CF patients aged 8 and 21 years was associated with an increase in the rate of lung function decline [33]. *Aspergillus* species are the most common filamentous fungi recovered from CF airways [15]. *Aspergillus* can exacerbate lung inflammation, establish infection and trigger hypersensitivity responses. *Aspergillus* can cause several clinical phenotypes in CF: *Aspergillus* colonization, *Aspergillus* bronchitis, *Aspergillus* sensitization, and allergic bronchopulmonary aspergillosis [34]. Gangell et al. investigated the inflammatory responses to individual microorganisms in the BAL fluids of pediatric CF patients (24 days to seven years old). They found that infection with *Pseudomonas aeruginosa*, *Staphylococcus aureus*, or *Aspergillus* was associated with significant inflammatory responses, as evidenced by the significantly increased neutrophil counts, increased free neutrophil elastase activity, and increased IL-8 levels [35]. (ii) Both Subjects A and B showed inconsistency in their treatments, which could contribute to the frequency of exacerbations and overall degree of illness. However, it doesn’t explain the differences between the two of them. And (iii) another possible reason could be the differences in their genetic backgrounds. Modifier genes and epigenetic changes have been shown to influence *CFTR* expression and cause phenotypic variability [12,36]. Wider genetic analysis (e.g., whole exome sequencing [37]) could reveal some differences that contribute to variations in their disease manifestation.

Our study has some limitations. Due to the low-volume sample size, we did not measure neutrophil counts or total cell counts as Mayer-Hamblett and colleagues did in an association study [21]. We did not have enough sample to run multiplex analysis from Subject B on Visit 1 when PE occurred. The lack of statistical significance in some of the analyses could be due to the small sample size. Furthermore, because this is a study of two subjects, cautions should be exercised when generalizing these findings to a larger CF population.

The development of CFTR-modulating drugs, including Kalydeco^®^, Orkambi^®^, Symdeko^®^, and Trikafta™ represents important milestones in the personalized medicine in CF, and has the potential to revolutionize CF care and management [38]. For individual patients, a better understanding of their disease history and identification of their specific biomarkers will help develop their personalized medical care. In this study, Subject A was taking Orkambi^®^ while Subject B was not on it because of drug–drug interaction issues. After our study, the subjects continued mostly the same therapy/treatment until they started triple combination CFTR modulators with Elexacaftor/Tezacaftor/Ivacaftor (Trikafta™) in late 2019. They experienced an improvement in lung function and reduced PEs. Both have now transitioned their care to an adult CF program. It will be interesting to see the long-term effect of Trikafta™ on their disease trajectories and how inflammatory mediators (e.g., NE and IL-8) respond to this novel therapy.

## 4. Materials and Methods

### 4.1. Subjects’ Characteristics

Subject A and Subject B received standard care at the University of Tennessee CF Research and Care Center at LeBonheur Children’s Hospital (Memphis, TN, USA). In this study, sputum samples were collected during their routine quarterly outpatient visits and during exacerbations, and processed according to standard procedures [39]. Their medical records and CF Foundation Registry data were analyzed retrospectively. The study was approved by the Institutional Review Board at the University of Tennessee Health Science Center (15-03851-XP). Written consent was obtained from parents of the study subjects. Only de-identified information is presented in this paper. The visits for Subject A were (month/year): Visit 1 (11/2015), Visit 2 (08/2016), Visit 3 (09/2016), Visit 4 (02/2017), Visit 5 (03/2017), Visit 6 (05/2017), Visit 7 (06/2017). For Subject B: Visit 1 (08/2016), Visit 2 (02/2017), Visit 3 (03/2017), Visit 4 (05/2017), Visit 5 (06/2017).

### 4.2. Measurements of IL-8 and NE Levels in Sputum Samples Using ELISA

IL-8 levels were measured using a Quantikine^®^ ELISA kit (D8000c, R&D, Minneapolis, MN, USA) following manufacturer’s instruction. The sensitivity of the kit is 7.5 pg/mL. NE levels were measured using a human PMN elastase ELISA kit (ab119553, Abcam, Cambridge, MA, USA) following manufacturer’s instruction. The sensitivity of the kit is 1.98 pg/mL. The detailed experimental procedures are provided in the Supplemental Materials.

### 4.3. ProcartaPlex™ Multiplex Immunoassay

The levels of a panel of inflammatory mediators in sputum samples were measured using an 18-plex ProcartaPlex™ Multiplex Immunoassay according to manufacturer’s instructions (Invitrogen, Carlsbad, CA, USA). Cytokine standards were prepared to determine the concentration of cytokines in the samples. The samples were run on a Millipore Magpix instrument (Burlington, MA, USA) and analyzed with xPONENT software (Version 4.2, Austin, TX, USA). For data analysis, a five-parameter logistic curve fitting method was applied to the standards and the sample concentrations extrapolated from the standard curve.

### 4.4. Statistical Analyses

To determine if there is a correlation between FEV_1_ and IL-8 levels, FEV_1_ and NE levels, two types of correlation coefficients, the product-moment (Pearson) and rank (Spearman), were calculated. The analysis was conducted using the R programming language (Version 3.4.0, The R Foundation, Vienna, Austria). Two-tailed *t*-test was used to compare the IL-8 and NE levels between two subjects. *p* < 0.05 was considered significant.

## Figures and Tables

**Table 1:** Pulmonary function test (PFT) results of Subject A and Subject B.

Visit #	Subject A							Subject B				
	1 *	2	3 ^†^	4	5	6	7	1 ^¶^	2	3	4	5
FEV_1_ (% predicated)	45	38	ND ^‡^	43	34	ND ^‡,§^	39	53	73	57	48	90
FVC (% predicated)	65	58	ND ^‡^	66	51	ND ^‡,‖^	61	64	87	69	64	97

#: Number. * Being admitted into the emergency room (ER) 6 days later for pulmonary exacerbation (PE). ^†^ Being admitted into ER 5 days later for PE. ^‡^ Unable to perform PFTs due to feeling too sick. ^§^ FEV_1_: 35% after 5 days antibiotics treatment. ^‖^ FVC: 50% after 5 days antibiotics treatment. ^¶^ Being admitted into ER for PE on this visit. Abbreviation: ND: no data available.

**Table 2:** Other major disease features of Subject A.

Visit #	1 *	2	3 ^†^	4	5	6	7
Major symptoms	Cough, low-grade fevers (99 °F).	Worsen symptoms, cough with green sputum.	Felt too sick to perform PFT.	Increased congestion, sore throat, and cough.	Productive cough, low-grade fevers.	Fever (102 °F), cough, diffuse crackles and expiratory wheeze.	Increased cough without fever.
Microbes in sputum cultures	3+ normal flora, 2+ MRSA, several *Aspergillus fumigatus*.	4+ MRSA, 4+ normal flora, 1+ *Stenotrophomonas maltophilia*, 2+ *Aspergillus fumigatus*.	No sputum culture sent.	No sputum culture sent.	4+ normal flora, 2+ MRSA.	2+ MRSA, 2+ normal flora, few *Stenotrophomonas maltophilia*, few *Aspergillus fumigatus*.	No sputum culture sent.

#: Number. * Being admitted into emergency room (ER) 6 days later for pulmonary exacerbation (PE). ^†^ Being admitted into ER 5 days later for PE. Abbreviation: MRSA: methicillin-resistant *Staphylococcus aureus*.

**Table 3:** Other major disease features of Subject B.

Visit #	1 *	2	3	4	5
Major symptoms	Increased productive cough, blackish-tinged sputum, low-grade fevers, and rhinorrhea.	Mild increase in cough, decreased air exchange and wheezing.	Increased cough, sputum production, congestion, and low-grade fevers.	Reported worsened cough and sputum production. Crackles heard in right base.	GI illness developed 3–4 days prior to visit.
Microbes in sputum cultures	2+ normal flora, 1+ non-mucoid *Pseudomonas aeruginosa*, 3+ MRSA, few *Stenotrophomonas maltophilia*.	No sputum culture obtained.	4+ normal flora, 2+ *Candida albicans*, several *E. Coli*, few MRSA.	1+ MRSA, few non-mucoid *Pseudomonas aeruginosa*, rare *Candida albicans*, few *Klebsiella pneumoniae*, 2+ normal flora.	No sputum culture obtained

#: Number. * Being admitted into the emergency room for pulmonary exacerbation on this visit. Abbreviations: GI: gastrointestinal; MRSA: methicillin-resistant *Staphylococcus aureus*.

**Table 4:** The average IL-8 and NE levels in the sputum samples from Subject A and Subject B.

	Subject A			Subject B		
	Average (Visit 1–7)	Non-PE (Visit 2, 4–7)	Preceding PE (Visit 1, 3)	Average (Visit 1–5)	Non-PE (Visit 2–4)	During PE (Visit 1)
IL-8 (pg/mL)	62,942	56,773	78,364	61,823	34,200	172,314
NE (ng/mL)	18.9	5.7	51.9	6.1	3	18.5

Abbreviation: PE: pulmonary exacerbation.

## Data Availability

The data presented in this study are available on request from the corresponding authors. The data are not publicly available due to privacy and ethical restrictions.

## Supplementary Materials

The following are available online at https://www.mdpi.com/1422-0067/22/5/2631/s1, Experimental procedure: Measurements of IL-8 and NE levels in sputum samples using ELISA.

## Abbreviations

BAL fluid	broncho-alveolar lavage fluid
CF	cystic fibrosis
CFTR	CF transmembrane conductance regulator
DAMP	damage-associated molecular patterns
ELISA	enzyme-linked immunosorbent assay
ER	emergency room
FEV_1_	forced expiratory volume in 1 s
FVC	forced vital capacity
GI	gastrointestinal
IL-	interleukin-
IP-10	IFNγ-induced protein 10 kDa
MRSA	methicillin-resistant staphylococcus aureus
NE	neutrophilic elastase
PE	pulmonary exacerbation
PFT	pulmonary function test
