# Supplementary material for: Two Siblings Homozygous for F508del-CFTR Have Varied Disease Phenotypes and Protein Biomarkers
Source: Int J Mol Sci. 2021 Mar 5;22(5):2631. doi: 10.3390/ijms22052631 (PMC7961721; doi:10.3390/ijms22052631)
Supplement: Supplementary file 1 [file ijms-22-02631-s001.pdf]

International Journal of *Molecular Sciences*

Supplemental Materials

Case Report

Two Siblings Homozygous for F508del-CFTR Have Varied Disease Phenotypes and Protein Biomarkers

Zhihong Zhang [1], Jin Wang [2], Yanhui H. Zhang [3], Tonia E. Gardner [1,4*], Elizabeth A. Fitzpatrick [2*] and Weiqiang Zhang[1,4-6*]

[1] Department of Pediatrics, College of Medicine, University of Tennessee Health Science Center, Memphis, TN 38163, USA; tgardn13@uthsc.edu (T.E.G.), wzhang16@uthsc.edu (W.Z.)
[2] Department of Microbiology, Immunology, and Biochemistry, College of Medicine, University of Tennessee Health Science Center, Memphis, TN 38163, USA; jwang141@uthsc.edu (J.W.), efitzpat@uthsc.edu (E.A.F.)
[3] Department of Bioscience Research, College of Dentistry, University of Tennessee Health Science Center, Memphis, TN 38163, USA; yzhang36@uthsc.edu
[4] University of Tennessee Cystic Fibrosis Care and Research Center at Le Bonheur Children's Hospital-Methodist University Hospital, Memphis, TN 38103, USA.
[5] Departments of Physiology, College of Medicine, University of Tennessee Health Science Center, Memphis, TN 38163, USA.
[6] Children's Foundation Research Institute, Le Bonheur Children's Hospital, Memphis, TN 38103, USA.
* Correspondence: (W.Z.); efitzpat@uthsc.edu (E.A.F.); tgardn13@uthsc.edu (T.E.G.)

Citation: Homozygous for F, T.S.; Biomarkers, P. Case Report. *Int. J. Mol. Sci.* **2021**, *22*, 2631. https://doi.org/10.3390/ijms22052631

Academic Editor: Stefanie Krick

Received: 7 February 2021
Accepted: 1 March 2021
Published: 5 March 2021

Publisher's Note: MDPI stays neutral with regard to jurisdictional claims in published maps and institutional affiliations.

Copyright: © 2021 by the authors. Submitted for possible open access publication under the terms and conditions of the Creative Commons Attribution (CC BY) license (http://creativecommons.org/licenses/by/4.0/).

Experimental Procedure

Measurements of IL-8 and NE levels in sputum samples using ELISA

IL-8 levels were measured using a Quantikine® ELISA kit (D8000c, R&D, Minneapolis MN, USA) following manufacturer's instruction. Briefly, Fifty microliters (50 μL) of standards and samples were added into appropriate wells containing 100 μL assay diluent, incubated at room temperature for 2 hours, followed by washing 4 times. One hundred microliters (100 μL) of IL-8 conjugate was added to each well and incubated at room temperature for 1 hour. After washing 4 times, two hundred microliters (200 μL) of substrate was added to each well and incubated at room temperature for 30 minutes in the dark. The stop solution was added to each well and the absorbance was read at 450 nm and 570 nm on a microplate reader (BioTek, Winooski, USA). IL-8 concentrations were calculated using the equation provided in the instruction. The sensitivity of the kit is 7.5 pg/mL.

NE levels were measured using a human PMN elastase ELISA kit (ab119553, Abcam, Cambridge MA, USA) following manufacturer's instruction. Briefly, the wells in a microplate were washed twice using the wash buffer. After removing the wash buffer, one hundred microliters (100 μL) of the prepared standards and samples were added into designated wells, incubated at room temperature for 1 hour on a shaker (400 rpm), and followed by washing 4 times. One hundred and fifty microliters (150 μL) of horseradish peroxidase (HRP)-conjugated antibody was added to each well and incubated at room temperature for 1 hour on a shaker (400 rpm). After washing 4 times, two hundred microliters (200 μL) of 3,3',5,5'-Tetramethylbenzidine (TMB) substrate solution was added to each well and incubated at room temperature for 20 minutes in the dark. Fifty microliters (50 μL) of the stop solution was added into each well and absorbance was read on the BioTek microplate reader at 450 nm and 620 nm. The NE concentrations were calculated using the equation provided in the instruction. The sensitivity of the kit is 1.98 pg/mL.
